# The impact of telomere length on the risk of idiopathic normal pressure hydrocephalus: a bidirectional Mendelian randomization study

**DOI:** 10.1038/s41598-024-65725-7

**Published:** 2024-06-26

**Authors:** Zhihao Wang, Mingrong Zuo, Wenhao Li, Siliang Chen, Yunbo Yuan, Yuze He, Yuan Yang, Qing Mao, Yanhui Liu

**Affiliations:** 1 Department of Neurosurgery, West China Hospital, Sichuan University, No 37 Guoxue Avenue, Chengdu, 610041 Sichuan China; 2 Department of Pediatric Neurosurgery, West China Women’s and Children’s Hospital: Sichuan University West China Second University Hospital, Chengdu, 610041 China

**Keywords:** Idiopathic normal pressure hydrocephalus, Telomere length, Mendelian randomization, Causality, Hydrocephalus, Epidemiology, Genetics research, Genetics

## Abstract

Idiopathic normal pressure hydrocephalus (iNPH) affects mainly aged populations. The gradual shortening of telomere length (TL) is one of the hallmarks of aging. Whereas the genetic contribution of TL to the iNPH is incompletely understood. We aimed to investigate the causal relationship between TL and iNPH through the Mendelian randomization (MR) analysis. We respectively obtained 186 qualified single nucleotide polymorphisms (SNPs) of TL and 20 eligible SNPs of iNPH for MR analysis. The result of MR analysis showed that genetically predicted longer TL was significantly associated with a reduced odd of iNPH (odds ratio [OR] = 0.634 95% Confidence interval [CI] 0.447–0.899,* p* = 0.011). The causal association remained consistent in multivariable MR (OR = 0.530 95% CI 0.327–0.860, *p* = 0.010). However, there was no evidence that the iNPH was causally associated with the TL (OR = 1.000 95% CI 0.996–1.004,* p* = 0.955). Our study reveals a potential genetic contribution of TL to the etiology of iNPH, that is a genetically predicted increased TL might be associated with a reduced risk of iNPH.

## Introduction

As a surgically curable disease in the central nervous system (CNS), idiopathic normal pressure hydrocephalus (iNPH) is highly likely to occur in aged populations and classically presented with an insidious clinical triad of dementia, gait disturbance, and urinary incontinence^1^. The reported morbidity of iNPH is not rare and is growing with increased age, with a prevalence of about 10 per 100,000 to 22 per 100,000 overall^2,3^. The representative radiographic manifestation of iNPH represents the formation of a ventriculomegaly, without cerebrospinal fluid pressure rising^4^. To alleviate the symptoms of iNPH, ventriculoperitoneal shunting has been designated the most effective treatment in approximately 60–80% of patients^5^. Unfortunately, it is miserable that about half of iNPH patients who experience initial improvement after shunt surgery may suffer from deterioration after more than 5 years of follow-up^6^. It hints that a better understanding of the underlying pathogenesis of iNPH is warranted. Unlike the secondary normal pressure hydrocephalus with known causes, the etiology and pathophysiological mechanism of iNPH remain to be elucidated^7^. Generally speaking, the pathophysiological features of iNPH are considered as follows: the impaired cerebral blood flow, the defective glymphatic system, and the dysfunction of the blood–brain barrier^7^. In addition, some previous observational research has illustrated that vascular risk factors, such as hypertension, diabetes mellitus, and overweight were risk factors for iNPH^8,9^. Even so, discordant findings in distinct studies suggest that potential causal factors correlated with iNPH need further clinical and experimental verification^9,10^. As iNPH is commonly found in the elderly, it is reported that about 10% of patients with dementia disorders can be diagnosed as iNPH and the population is expected to be over 150 million by 2050^11^. It warrants mention that iNPH and Alzheimer’s disease (AD) display somewhat overlapping symptoms^12^. To date, it is likely to misdiagnose these two disorders as no specific biomarker is available to differentiate iNPH and aging-related diseases. Given shared clinical features with AD, such as amyloid beta deposition, mislocalization of aquaporin-4 (AQP-4), cerebrovascular inflammation, and sleep disturbance^11^, probing underlying biomarkers for the diagnosis of iNPH has been in crying need.

Telomeres located at the end of the chromosomes can protect the genome from damage^13^, which gradually shortens over time in most normal cells as one of the hallmarks of aging^14^. Telomere attrition or loss, a process causally linked with cell senescence late in life caused by chromosomal instability, has been known to contribute to aging^15^. Additionally, accumulating evidence also suggests that telomere length (TL) is an effective predictor for multiple disorders. Clinically, shorter TL is associated with unhealthy physical conditions, such as smoking, increased body weight, and physical inactivity^16^. Shorter TL is also clearly tied to an increased risk for cardiovascular diseases^17^, type 2 diabetes^18^, and AD^19^. Regarding the genetic contribution to the development of congenital hydrocephalus, it suggests that X-linked hydrocephalus genes are associated with congenital hydrocephalus, and these genes with a high mutation rate are associated with proximity to telomeres and high adenine and thymine content^20^. More than 100 genes are reported to be capable of causing congenital hydrocephalus, for example, the zinc finger CCHC-type domain containing 8 protein (ZCCHC8), which is located in close vicinity of its telomere (< 50 Mbp) on chromosome 12^21^. Hence, genes near telomeres with high mutation rates may participate in the formation of congenital hydrocephalus. Whereas the association between TL and the iNPH is unknown.

Mendelian randomization (MR) analysis is less susceptible to confounding factors and the reverse causations that can impede causal inference, compared with conventional observational studies^22^. Thus, accumulating evidence suggests that MR is another momentous approach to investigating the causal relationship compared to randomized clinical trials. Although several theories have been demonstrated to be associated with the development of iNPH^1^, the causal factors of iNPH have rarely been investigated. A recent MR study found that essential hypertension was a causal risk factor for iNPH^10^, which was in line with previous research suggesting a correlation between hypertension and iNPH^8^. It suggested that longer TL is inversely correlated with dementia risk, including AD^19^. Due to both AD and iNPH being aging-related diseases and commonly being misdiagnosed, ascertaining the interrelationship of TL and iNPH may be available for directing research on the diagnosis and intervention strategies for iNPH.

Based on the summarized data of Genome-Wide Association Study (GWAS), the aim of the present study focused on the causal association of TL and iNPH through a bidirectional two-sample MR analysis, using germline genetic variants as instrumental variants (IVs). Our findings could provide more evidence on the pathogenesis of iNPH.

## Materials and methods

### Data sources

We obtained summary-level data on TL from the OpenGWAS database (GWAS ID: ieu-b-4879, https://gwas.mrcieu.ac.uk/) with 472,174 European participants in the UK Biobank^23^. TL of peripheral blood leukocytes was extracted with validated quantitative polymerase chain reaction methodology. A ratio of the telomere repeat number to the single-copy gene was applied as the measurement of TL^24^. Next, the GWAS data of iNPH were obtained from the European cohort: the FinnGen study^25^. The FinnGen study committed to combining genomic data with digital healthcare data^26^. The criterion in FinnGen was based on the 10th edition of the International Classification of Diseases, in which patients with a hospitalization history diagnosed as G91.2 were identified as NPH. In the FinnGen study round 9, a total of 767 iNPH cases and 375,610 controls were enrolled in the dataset. To conduct multivariable MR (MVMR), potential vascular risk factors of iNPH were included. GWAS for body mass index (BMI) (454,884 individuals) and primary hypertension (54,358 cases and 408,652 controls) were obtained from the Medical Research Council Integrative Epidemiology Unit (MRC-IEU) consortium^27^, with data from European participants in the UK biobank derived by PHESANT^28^. For type 2 diabetes (T2D), a GWAS composed of 62,892 T2D cases and 596,424 controls was employed, with most of the participants being European^29^.

**Figure 1:**
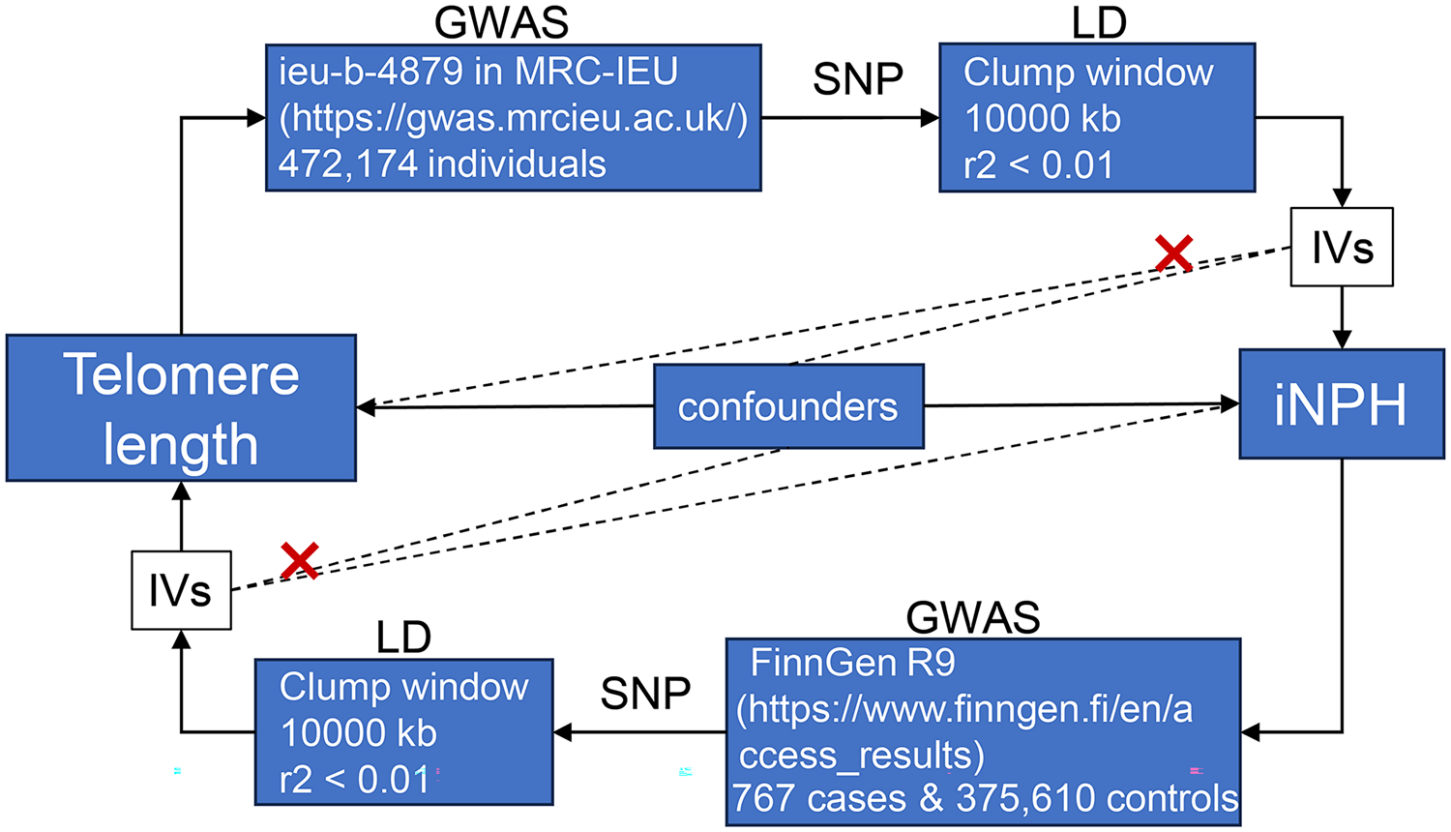
Design of the study. This figure illustrated detailed procedures of MR, which included three principal assumptions. (I) IVs must be associated with exposure. IVs cannot be associated with confounders (II) and outcomes (III). *MR* Mendelian randomization, *GWAS* Genome-wide association studies, *SNP* single nucleotide polymorphism, *LD* linkage disequilibrium, *iNPH* idiopathic normal pressure hydrocephalus, *IVs* instrumental variants.

We plotted the study with three principal assumptions of MR (Fig. 1). Firstly, there existed a direct correlation between the IVs of exposure (Relevance). Secondly, the confounders were unable to interfere with the IVs (Exchangeability). Lastly, the IVs had no direct connection to the outcome (Exclusion restriction).

### IVs selection

To select the qualified IVs based on the three assumptions of MR analysis, we adopted the criteria for IVs inclusion which required the genome-wide threshold (*p* < 5E-08). A less strict threshold of *p* < 5E−06 was applied if IVs were insufficient for sensitivity analysis. Single nucleotide polymorphisms (SNPs) with linkage disequilibrium (LD) were measured with European ancestry 1000 Genomes LD reference panel, within a 10,000 kb clump window, r2 > 0.01 was considered as LD and further adjusted to ensure independent IVs. To avoid bias of weak instrument, SNP with a low F-statistic (< 10) was to be removed. SNPs should be excluded if which was significantly associated with outcome (*p* < 5E−08) as inconformity with the third principle of MR assumption. SNPs that did not exist in outcome GWAS were deleted, with harmonization carried out in the remaining SNPs to control the match of alleles^30^. To attenuate bias due to rare variants, IVs with effect allele frequency less than 0.01 in exposure or outcome were eliminated. To validate the results of TL to iNPH, another set of IVs provided by Codd et al.^31^ was used as sensitivity analysis.

### Mendelian randomization

We applied the inverse variance weighted (IVW) analysis as primary method to obtain causal estimates between TL and iNPH. By using the random effect model, IVW allowed the existence of heterogeneity in the causal inference^32^. The simple mode and weighted mode were two mode-based estimation methods, sensitive to addition or removal of genetic variants, where simple mode was more biased than the weighted mode^33^. Weighted median model provides valid estimates despite up to half of invalid IVs by ordering the IVs according to their magnitude estimates^34^. Cochran’s Q statistics were generated by both the IVW and MR-Egger method, which was used to appraise the heterogeneity of IVs^35^, and *p* < 0.05 meant the existence of heterogeneity. The pleiotropy test was conducted with the intercept MR-Egger regression, which was sensitive to violations of Exclusion restriction assumption, with *p* > 0.05 representing no horizontal pleiotropy^36^. Mendelian Randomization Pleiotropy Residual Sum and Outlier (MR-PRESSO) was used to screen for outliers. For positive results in IVW, MVMR was conducted to adjust the potential influence of confounders, in which IVs should be associated with at least one of the exposures and independent of both the confounders and outcome^37^. The same inclusion criteria of IVs were executed in MVMR.

### Statistical analysis

Results were displayed as the OR and 95% CIs of iNPH risk and a unit standard deviation (SD) change in TL. Scatter plots were utilized for the visualization of MR results. Statistical analyses were performed using R-4.2.3 (https://www.R-project.org/) with R packages “TwoSampleMR”^30^ version 0.5.7 (https://github.com/MRCIEU/TwoSampleMR) and “MRPRESSO”^38^ version 1.0 (https://github.com/rondolab/MR-PRESSO). A two-sided *p* < 0.05 was considered a significant association between exposure and outcome.

### Ethics approval

No original data was generated in present study, summary-level statistics from published studies and publicly available data were reviewed by relevant institutional review board.

## Results

### The causal effect of TL on iNPH.

**Figure 2:**
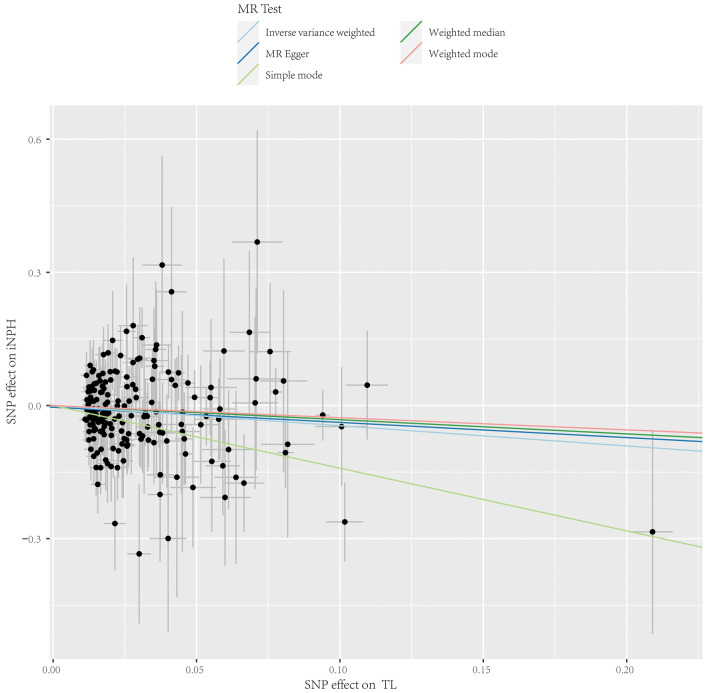
Scatter plots for MR analysis of the causal effect of TL on iNPH. Each dot represents a single SNP, and the line on each point means 95% CI. The slope of each line shows the MR results for the corresponding method. *MR* Mendelian randomization, *TL* telomere length, *iNPH* idiopathic normal pressure hydrocephalus, *SNP* single nucleotide polymorphism.

There were 191 qualified SNPs for TL at the genome-wide threshold. Notably, 30 palindromic SNPs were identified, in which five SNPs (rs2276182, rs2306646, rs4766578, rs56178008, and rs670180) were removed for being palindromic with intermediate allele frequencies. Thus, 186 SNPs were used in MR analysis (Supplementary Table S1). The MR analysis performed with the random effect IVW model revealed that TL was an effective causal factor for the risk of iNPH (OR = 0.63, 95% CI 0.45–0.90, *p* = 0.011) (Table 1), the same direction of effect was also presented in the rest four methods, indicating robust casual effect (Table 1 and Fig. 2). Specifically, heterogeneity estimation performed with IVW analysis (*p* = 0.453) and MR-Egger regression (*p* = 0.437) showed no IVs of TL was heterogeneous. In addition, the pleiotropy test did not find horizontal pleiotropy in MR analysis (*p* = 0.664). No significant outliers were detected by MR-MRESSO. Results of sensitivity analysis was displayed in Table 2.

**Table 1:** MR analysis of TL on iNPH.

Method	nSNP	OR (95% CI)	P value
Inverse variance weighted	186	0.634 (0.447, 0.899)	0.011
MR Egger	186	0.710 (0.381, 1.323)	0.283
Weighted median	186	0.726 (0.394, 1,338)	0.305
Simple mode	186	0.244 (0.067, 0.876)	0.032
Weighted mode	186	0.760 (0.367, 1,575)	0.461

*MR* Mendelian randomization, *TL* telomere length, *iNPH* idiopathic normal pressure hydrocephalus, *SNP* single nucleotide polymorphism, *OR* odds ratio, *CI* confidence interval.

**Table 2:** Sensitivity analysis of TL on iNPH.

Method	Measurement		P value
MR Egger heterogeneity	Cochrane Q (df)	186.402 (184)	0.437
IVW heterogeneity	Cochrane Q (df)	186.593 (185)	0.453
Pleiotropy	Intercept	− 0.004	0.664
Outliners	No significant outliers		

*TL* telomere length, *iNPH* idiopathic normal pressure hydrocephalus, *MR* Mendelian randomization, *IVW* inverse variance weighted.

Next, we included BMI, hypertension, and T2D as exposures for the MVMR analysis in investigate whether the effect of TL on iNPH was mediated through these potential confounders. After ruling out the SNPs that directly associated with iNPH, BMI, hypertension and T2D, 89 SNPs were identified as IVs for TL, the same screening process was applied in the IVs selection of other three exposures. Interestingly, it demonstrated that TL still had a significant negative association with the risk of iNPH (OR = 0.530, 95% CI 0.327–0.860, *p* = 0.010), while the rest of the three exposures showed no causal effect on iNPH (Table 3). Taken together, a genetically predicted increase in TL was associated with a lower risk of iNPH, independent of BMI, hypertension, and T2D.

**Table 3:** MVMR analysis of four exposures on iNPH.

Exposure trait	nSNP	OR (95% CI)	P value
TL	89	0.530 (0.327, 0.860)	0.010
BMI	487	0.903 (0.613, 1.332)	0.608
Hypertension	36	4.571 (0.254, 82.231)	0.303
T2D	93	1.064 (0.900, 1.258)	0.469

*MVMR* multivariable Mendelian randomization, *iNPH* idiopathic normal pressure hydrocephalus, *SNP* single nucleotide polymorphism, *OR* odds ratio, *CI* confidence interval, *T2D* type 2 diabetes, *TL* telomere length, *BMI* body mass index.

For duplicated sensitivity analysis, 197 SNPs reported by Codd et al. were enrolled, in which 130 variants were used for MR analysis. After mapping, 93 SNPs matched in iNPH GWAS, while five were dropped as their effect allele frequency less than 0.01. Another five were removed as they were palindromic with intermediate allele frequencies. Finally, 83 IVs were used for another round of sensitivity analysis (Supplementary Table S2). As shown in Supplementary Table S3, although IVW method did not found significant results (OR = 0.530, 95% CI 0.327–0.860, *p* = 0.338), all five methods suggested the same negative direction of effect. Neither heterogeneity nor pleiotropy was reported, and no outliners were found. Nevertheless, future studies might provide new insights.

### The causal effect of iNPH on TL

**Figure 3:**
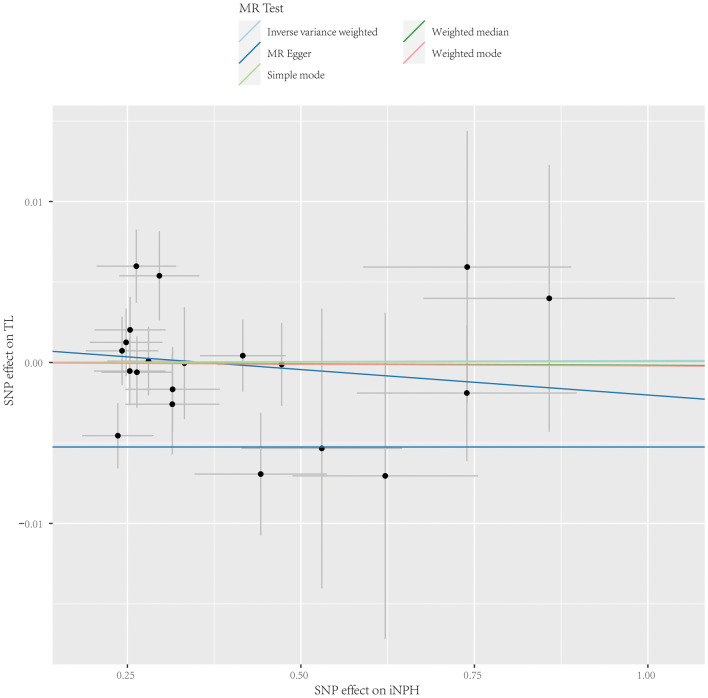
Scatter plots for MR analysis of the causal effect of iNPH on TL. Each dot represents a single SNP, and the line on each point means 95% CI. The slope of each line shows the MR results for the corresponding method. *MR* Mendelian randomization, *TL* telomere length, *iNPH* idiopathic normal pressure hydrocephalus, *SNP* single nucleotide polymorphism.

We also investigated the inverse causal effect of iNPH on TL. For IVs of iNPH, A threshold of *p* < 5E−06 was adopted to hire sufficient IVs for sensitivity analysis.None of the 20 SNPs was palindromic, with all F-statistics above 20 (Supplementary Table S4). The result of the IVW analysis revealed no suggestive evidence of a causal relationship of iNPH on TL (Table 4 and Fig. 3). In addition, no heterogeneity and horizontal pleiotropy existed (Table 5).

**Table 4:** MR analysis of iNPH on TL.

Method	nSNP	OR (95% CI)	P value
Inverse variance weighted	20	1.000 (0.996, 1.004)	0.955
MR Egger	20	0.997 (0.985, 1.009)	0.624
Weighted median	20	1.000 (0.995, 1.005)	0.946
Simple mode	20	1.000 (0.992, 1.008)	0.991
Weighted mode	20	1.000 (0.993, 1.006)	0.949

*MR* Mendelian randomization, *iNPH* idiopathic normal pressure hydrocephalus, *TL* telomere length, *SNP* single nucleotide polymorphism, *OR* odds ratio, *CI* confidence interval.

**Table 5:** Sensitivity analysis of iNPH on TL.

Method	Measurement		P value
MR Egger heterogeneity	Cochrane Q (df)	23.123 (18)	0.186
IVW heterogeneity	Cochrane Q (df)	23.507 (19)	0.216
Pleiotropy	Intercept	0.001	0.216
Outliners	No significant outliers		

*iNPH* idiopathic normal pressure hydrocephalus, *TL* telomere length, *MR* Mendelian randomization, *IVW* inverse variance weighted.

## Discussion

In our study on the causal association of TL with iNPH, we demonstrated that genetically predicted TL was thought to be associated with iNPH and that a longer TL reduced the risk of iNPH, which remained significant after adjusting the potential confounding effects of BMI, hypertension, and T2D. Replicated sensitivity analysis also revealed no potential violation of MR assumptions, though not significant results were reported by IVW methods. In turn, there was no suggestive causal effect of iNPH on the TL. Thus, our study shed fresh light on identifying TL as a potential causal factor associated with iNPH, which should be interpreted with caution.

Our understanding of the pathophysiology of iNPH has evolved considerably in recent years. The imbalance of production and absorption of cerebrospinal fluid (CSF) was conjectured to be the main reason for iNPH. For example, obstruction of the arachnoid granulations would impair CSF returning to circulation^39^. Another piece of evidence challenged this theory by observing that subarachnoid spaces were not always ectatic in communicating hydrocephalus, which suggested that a new equilibrium may be reached in CSF circulation^39^. As there was no apparent lesion causing the restricted CSF outflow in iNPH, impaired vascular compliance was considered another pathogenic factor for iNPH^40^. Cardiac pulsations transferred through arterial vessels propelled CSF movement in a craniocaudal way in health conditions^40^, however, the net movement was reversed in iNPH patients^40^. Phillip A. Bonney et al. equated the development of iNPH with a cerebrovasculature disorder^41^ as the decreased compliance of the cardiovascular system caused by hypertension or diabetes may alter CSF dynamics culminating in the formation of hydrocephalus^8,42^. Due to inconsistent results of the vascular risk factors of iNPH produced by distinct studies, a recent meta-analysis investigated the effect of multiple reported vascular risk factors on iNPH and found that hypertension, diabetes mellitus, coronary heart disease, peripheral vascular disease, and overweight were risk factors of iNPH^9^. Interestingly, a recent MR study demonstrated that essential hypertension was a causal risk factor for iNPH compared to other factors that showed no causal relationship, such as T2D and overweight^10^. It warrants noting that our MVMR results support TL as a potential causal factor of iNPH after adjusting vascular risk factors, such as T2D, hypertension, and BMI. As existing inconsistent causal factors for iNPH, we should be prudent with each finding due to the following reasons: (i) The limited sample size of iNPH in the FinnGen study and distinct statistical methods may result in inconsistent results, (ii) The complicated mechanisms of iNPH are tough to be explained by existing approaches. Based on these findings, it is essential to rediscover the role and pathophysiological mechanism of vascular risk factors and TL in forming iNPH by enlarging the cohort sizes.

Deficient cerebral blood flow (CBF) was commonly found in multiple areas of the brain of iNPH patients as compared to the age-matched healthy controls^43^. The glymphatic (glial-lymphatic) system in the CNS also played a vital role in removing toxoids and waste products by draining the CSF out of the brain^44^. iNPH patients were likely to have a sluggish glymphatic flow^45^, which was also involved in the AD^46^. One supposed theory for iNPH was a loss of arterial compliance which could weaken the glymphatic influx and result in CSF retention^41^. Another verified mechanism for glymphatic impairment related to decreased expression of AQP-4, which promoted the removal of superfluous fluid and waste metabolites from CNS^47^. The blood–brain barrier (BBB) was pivotal in maintaining the homeostasis of the CNS. Impaired BBB was reported to be associated with iNPH. For example, the degenerated pericyte processes were found in biopsy samples from iNPH patients^48^. This alteration has been shown to cause increased fluid permeation. In addition, the breakdown of BBB could also promote the extravasation of fibrin in the brain of iNPH patients^48^. Increased fibrin in the brain of iNPH patients was associated with astrogliosis, which could reduce compliance of vessels in the brain parenchyma and may lead to altered CSF dynamics^48^. Taken together, the present perception of the pathogenesis of iNPH is closely involved in the cerebrovascular system. Future studies should explore the specific molecular mechanisms of deficient CBF, glymphatic impairment, and changes to the BBB in regulating the development of iNPH.

Regarding the genetically etiological mechanisms of iNPH, it has been so far mostly unknown. Recently, a critical study unveiled the mechanisms of iNPH, which found that 15% of iNPH patients with two recurrent heterozygous loss of function deletions in CWH43. Heterozygous CWH43 deletion induced mice to develop enlarged ventricles, gait abnormality, and decreased cilia numbers^49^. These findings provided fresh mechanistic insights into the origins of iNPH. Another study defined a loss-of-function variant in the CFAP43 gene related to NPH in one Japanese family by performing whole-exome sequencing^50^. Due to CFAP43 encoding cilia- and flagella-associated protein, mice with CFAP43 knock-out exhibited hydrocephalus with an abnormality of motile cilia^50^. It was rational to speculate that cilia played an important role in maintaining CSF circulation, genetic abnormalities of cilia were key molecular mechanisms of iNPH. As loss of AQP-4 expression is significantly presented in the astrocytic endfoot membranes in iNPH patients^51^, and telomere attrition could induce astrocyte apoptosis in age-related neurodegenerative diseases^52^, future studies should probe the mechanisms of cilia and astrocytes in mediating the development of iNPH. The genetic connection between iNPH and other neurological diseases has also been studied. One study revealed none of AD-associated SNPs had significant effect on the accumulation of amyloid-β in the brain of iNPH patients, but APOE4 was associated^53^. Another study found NME8-related SNP rs2718058, whose genotype (AA) was risk factor of AD, was associated with iNPH independent of known AD pathology^54^. All these evidences suggested iNPH was an independent disease entity.

In our MR study, we provided novel insight into the etiology of iNPH by demonstrating that genetically predicted shorter TL was a potential causal factor for the risk of iNPH. TL was proven to be associated with multiple disorders, such as aging-related diseases and neoplasms. Emerging studies have tried to target telomeres for cancer treatment by regulating the telomere-maintenance mechanism, which mainly includes telomerase or the alternative lengthening of telomeres pathway^55^. Regarding the role of telomeres in diagnosing hydrocephalus, one pilot study found that transient receptor potential cation channel subfamily V member 4 (TRPV4) mRNA, proximity to telomeres, was upregulated in the caudate nucleus tissues of NPH patients, which expressions were conserved across other two species of mice and rats^56^. Whereas the expression of TRPV4 was not changed in AD patients, suggesting that TRPV4 may be applicable to differentiate diagnoses and a large cohort study should be conducted to investigate the transformation value in diagnosing NPH^56^. Another research investigating the genetic etiology of congenital hydrocephalus mentioned that 98 of 108 genes causing congenital hydrocephalus conformed to the following two criteria: (i) proximity to telomeres or (ii) high adenine and thymine content^21^. Moreover, it suggested that TL was associated with the status of astrocytes^52^. Thus, we raised a presumption that genetic mutations near telomeres, which functionally regulate the alteration of TL, may participate in the genesis of iNPH. The mechanism of TL in the development of iNPH needs further investigation to seek out the mechanisms of iNPH evolution.

Our study has several deficiencies. First, most of the participants involved in this study were European, which meant our findings may not apply to other ethnic groups. Second, we did not conduct an external verification as only one GWAS data of iNPH was available. Nonetheless, we utilized multiple approaches to validate the robustness of the causal relationship, such as using the MR egger and weighted median analysis. Third, despite the IVW results in replicated sensitivity analysis did not demonstrate significant causality, the same direction of effect was revealed. Thus, our results must be interpreted with caution, further researches are warranted for more conclusive results.

## Conclusion

In conclusion, our MR analysis provided evidence of the potential causal effect of shorter TL on the risk of iNPH. This suggested TL may be one of the pathophysiological mechanisms of iNPH. The specific mechanisms of TL participating in the development of iNPH are worth discussing in the near future and a larger clinical cohort is required to certify our results. Our novel evidence may be conducive to directing research on the diagnosis and intervention strategies for iNPH.

### Supplementary Information

Supplementary Tables.

## Data Availability

The original data of our study was from public datasets. Summarized GWAS data for TL (ieu-b-4879)^23^, hypertension (ukb-b-12493), and BMI (ukb-b-2303) were downloaded via the MRC-IEU OpenGWAS database (https://gwas.mrcieu.ac.uk/)^27^. GWAS of T2D was obtained from Xue et al.^29^. GWAS of iNPH was from FinnGen Release 9 (https://www.finngen.fi/en/access_results)^26^. IVs for replicated analysis were available in Supplementary Table S1 of original study^31^.
